# Non-ergodic extended regime in random matrix ensembles: insights from eigenvalue spectra

**DOI:** 10.1038/s41598-023-27751-9

**Published:** 2023-01-12

**Authors:** Wang-Fang Xu, W. J. Rao

**Affiliations:** 1 School of Science, Hangzhou Dianzi University, Hangzhou, 310027 China; 2 China Academy for Rural Development and School of Public Affairs, Zhejiang University, Hangzhou, 310027 China

**Keywords:** Phase transitions and critical phenomena, Phase transitions and critical phenomena

## Abstract

The non-ergodic extended (NEE) regime in physical and random matrix (RM) models has attracted a lot of attention in recent years. Formally, NEE regime is characterized by its fractal wavefunctions and long-range spectral correlations such as number variance or spectral form factor. More recently, it’s proposed that this regime can be conveniently revealed through the eigenvalue spectra by means of singular-value-decomposition (SVD), whose results display a super-Poissonian behavior that reflects the minibands structure of NEE regime. In this work, we employ SVD to a number of RM models, and show it not only qualitatively reveals the NEE regime, but also quantitatively locates the ergodic-NEE transition point. With SVD, we further suggest the NEE regime in a new RM model–the sparse RM model.

## Introduction

The physics in isolated quantum systems has attracted a lot of attention in current condensed matter society, where people have established the existence of two generic quantum phases: the ergodic phase that obeys the eigenstate thermalization hypothesis, and a many-body localized (MBL) phase where interaction and localization coexist^1–3^. The traditional way to distinguish the ergodic/MBL phase is by studying their eigenvalue statistics, whose mathematical foundation is the random matrix (RM) theory^4,5^. Specifically, eigenvalues in ergodic phase are well-correlated and fall into the Wigner–Dyson class, while MBL phase has uncorrelated eigenvalues that follow the Poisson ensemble^6–13^. Modern understanding is through the quantum entanglement: the entanglement in ergodic phase is extensive that scales linearly with system’s size (the volume-law), while a MBL phase holds small (area-law) entanglement^14–20^.

Compared to the two individual phases, much less is known about the ergodic-MBL transition. This is partially due to the intrinsic numerical difficulty in studying non-equilibrium systems, that is, we in principle have to compute all the eigenvalues (or at least a finite portion of them) to fully describe the phases, hence the computational cost grows exponentially with system’s size. Despite the worthwhile attempt to approach larger computational resources, people have also been searching for new methods to explore the hidden physics from the eigenvalue spectra, which is technically much easier to obtain than the wavefunctions.

Recently, an innovative approach is to view the eigenvalue spectra of disordered quantum systems and RM models as multi-variant time series^21–28^, and by the data-adaptive technique of singular value decomposition (SVD), we are able to study the novel phenomena of non-ergodic extended (NEE) regime. The NEE regime is a finite region locating between the ergodic and MBL phases, wwhich is characterized by a clustering of eigenvalues that forms the so-called miniband structure, and the eigenvalue correlations in the same miniband are much stronger than those between different bands. As a result, the eigenvalues follows a super-Poissonian behavior^29,30^ that can be conveniently revealed through the SVD results. This method has been applied to the Rosenzweig-Porter (RP) model with known NEE regime, the Anderson model and the random field Heisenberg models^30–33^. Compared to traditional eigenvalue-based probes of NEE regime such as the number variance or spectral form factor^34–37^, SVD does not require the cumbersome unfolding procedure that is non-unique and may cause extra confusions^38^.

Given the efficiency of SVD method, an important question still remains: Is SVD only a qualitative way to reveal the existence of NEE regime, or can it provide a quantitative estimation for the boundary of NEE regime? Moreover, can we use SVD to search for NEE regime in unknown RM models? In this work we aim to answer these two questions simultaneously by applying SVD to a number of RM models.

This paper is organized as follows. In “SVD on power-law random banded matrix ensemble” we introduce the method of SVD and apply it to the power-law random banded matrix (PRBM) ensemble, which is another known RM model besides the RP model that holds NEE regime^39–42^. We will discuss in detail about the mechanism of SVD and show that SVD not only reveals the existence of NEE regime, but also provides an accurate estimation for the ergodic-NEE transition point. In “Scree plots of other RM models” we employ SVD to three related RM models: (i) the RP model, where a more detailed study shows the ergodic-NEE transition point is also captured by SVD; (ii) a sparse RM model that describes the percolation between Wigner–Dyson and Poisson ensemble, where a new NEE regime is observed; (iii) the Gaussian $\beta$ ensemble, where no super-Poissonian behavior exists. To show the generalizability of this method, we also apply SVD to the empirical data from stock market and obtain interesting results, which will be presented in the supplementary material S1. Conclusion and discussion are given in “Conclusion and discussion”.

## SVD on power-law random banded matrix ensemble

The first RM model we consider is the power-law random banded matrix (PRBM) ensemble^43^, which is a Gaussian ensemble of $D_{H}\times D_{H}$ symmetric matrices *H* with random elements, whose distribution satisfy

1 $$\begin{aligned} \langle H_{ij}\rangle =0\text {, }\langle \left( H_{ij}\right) ^{2}\rangle =\beta ^{-1}\left[ 1+\left( \left| j-i\right| /B\right) ^{2\mu } \right] ^{-1} \end{aligned}$$

where $H_{ij}$ are real random elements, and $\mu \in \left( 0,\infty \right)$ is the tuning parameter. In this study we focus on the orthogonal PRBM with $\beta =1$ and fix $B=1$ without loss of generality. It’s known that PRBM exhibits a metal-insulator transition at $\mu _{c}=1$^43–46^, while recent studies establish the NEE regime in the intermediate range $0.5<\mu <1$ by evaluating its wavefunctions’ multi-fractal scaling^40,41^, and here we aim to confirm it from the eigenvalue point of view with SVD.

To do SVD, we diagonalize $N=1000$ samples of PRBMs with matrix dimension $D_{H}=8000$ at various $\mu$s to obtain the eigenvalue spectra $\left\{ E_{i}\right\}$, and select $P=2000$ eigenvalues in the middle of each sample to construct the following $N\times P$ matrix *X*,

2 $$\begin{aligned} X=\left( \begin{array}{cccccc} E_{1}^{\left( 1\right) } &{} E_{2}^{\left( 1\right) } &{} . &{} . &{} . &{} E_{P}^{\left( 1\right) } \\ E_{1}^{\left( 2\right) } &{} E_{2}^{\left( 2\right) } &{} . &{} . &{} . &{} E_{P}^{\left( 2\right) } \\ . &{} . &{} . &{} &{} &{} . \\ . &{} . &{} &{} . &{} &{} . \\ . &{} . &{} &{} &{} . &{} . \\ E_{1}^{\left( N\right) } &{} E_{2}^{\left( N\right) } &{} . &{} . &{} . &{} E_{P}^{\left( N\right) } \end{array} \right) \text {,} \end{aligned}$$

where $E_{i}^{\left( j\right) }$ stands for the *i*-th eigenvalue in the *j*-th sample, and we shall call *X* the “sample matrix” throughout this paper. We then perform SVD on *X*, which equals to decompose *X* into

3 $$\begin{aligned} X=U^{T}\Lambda W\equiv \sum _{k=1}^{r}\sigma _{k}X^{\left( k\right) }\text {, } X_{ij}^{\left( k\right) }=U_{ik}^{T}W_{kj}\text {,} \end{aligned}$$

where $\sigma _{i}$ are the ordered singular values $\sigma _{1}\ge \sigma _{2}\ge \cdots \ge \sigma _{r}$ with $r\le \min [N,P]=$ Rank[*X*]. This technique is in fact equivalent to the machine learning algorithm called principal component analysis (PCA), the spirit of which is to view the eigenvalue spectra as a multi-dimensional data, and by SVD we decompose it into orthonormal components represented by $W_{k}$ – the *k*-th row of the $P\times P$ matrix *W* – with weight $\sigma _{k}$. It is known the scree plot – $\lambda _{k}=\sigma _{k}^{2}$ as a function of index *k* – behave differently in different phases, namely $\lambda _{k}\sim k^{-\alpha }$ with $\alpha =1(2)$ in ergodic (localized) phases^21,22^, representing the chaotic (integrable) behaviors. The power-law behaviors indicate the long-range spectral correlations are scale-invariant (fractal)^23,24^.

**Figure 1:**
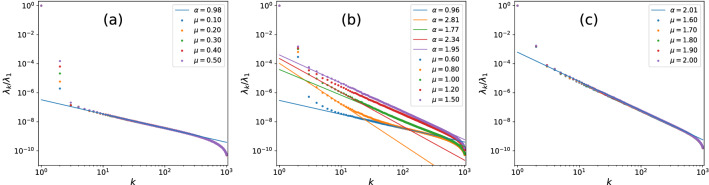
Scaled scree plots of $\left\{ \lambda _{k}/\lambda _{1}\right\}$ of PRBM at various parameters $\mu$. Despite the two dominant modes, $\lambda _{k}\left( k\ge 3\right)$ follows a power-law $k^{-\alpha }$ with $\alpha \simeq 1$ for $\mu \le 0.5$ and $\alpha \simeq 2$ for $\mu \ge 1.6$, representing chaotic and integrable regimes respectively. In the intermediate range $\mu \in \left( 0.5,1.6\right)$, two-branch scree plots appear with the lower part displays a super-Poissonian behavior $\alpha >2$, indicating a NEE regime. The two-branch structure starts to appear at $\mu =0.5$, in consistent with the value predicted in Ref.^40^.

The scree plots of PRBM at various $\mu$s are presented collectively in Fig. 1, where we have plotted the scaled singular values $\left\{ \lambda _{k}/\lambda _{1}\right\}$ for eye’s convenience without affecting its scaling behavior. As we can see, in all cases, the first two weights $\lambda _{1/2}$ are orders of magnitudes larger than the rest, which stand for two non-universal features of the eigenvalues. While $\lambda _{k}$ with $k\ge 3$ further divides into three categories: (i) for $\mu \le 0.5$ the scree plots stays almost identical, following the chaotic behavior $\lambda _{k}\sim k^{-1}$, which clearly stand for the ergodic phase; (ii) when $\mu$ grows beyond 0.5, the scree plots begins to exhibit two-branch structures that both follows power-law, for reasons detailed below, this regime is identified to be the NEE regime; (iii) when $\mu \ge 1.6$, PRBM enters into the fully integrable regime with $\lambda _{k}\sim k^{-2}$.

**Figure 2:**
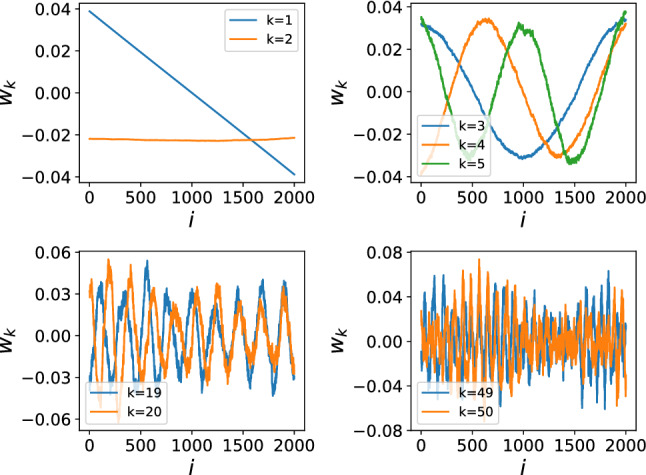
Behaviors of representative components $W_k$, the first two dominant modes $W_{1/2}$ are non-fluctuating, representing two non-universal features of the eigenvalue spectrum. Higher components with $k\ge 3$ are close to the *k*th Fourier modes of the eigenvalues, therefore, components with smaller (larger) *k* describes level correlations on longer (shorter) ranges.

To understand the physics of the scree plots, we must look into the detailed structures of the $W_{k}$. The analysis below is based on PRBM with $\mu =0.8$, and we have checked that they hold in other cases as well. Several typical $W_{k}$ are drawn collectively in Fig. 2, where the horizontal coordinate stands for the eigenvalue index *i*. Clearly, the first two components $W_{1/2}$ with dominant weights are both linear, which means the eigenvalue spectrum is dominated by two non-fluctuating features, the most natural guess of which would be the mean energy $\langle E\rangle$ and level spacing $\langle s\rangle$, both of which depend on the model’s details and hence are not universal. While $W_{k}$ with $k\ge 3$ behave closely to a quasi-sinusous function which means $W_{k}$ is close to the *k*-th Fourier modes of the eigenvalues, and therefore higher mode has shorter wave-length that describes level correlations on shorter-ranges.

To support above arguments, we proceed to study the power-spectrum functions. Here we consider two kinds of power-spectrum functions, both of them have appeared in previous studies. The first one is^30,31,47,48^

4 $$\begin{aligned} F\left( k\right) =\frac{1}{N}\sum _{m=1}^{N}F_{m}\left( k\right) \end{aligned}$$

where

5 $$\begin{aligned} F_{m}\left( k\right) =\left| \frac{1}{r}\sum _{n=1}^{r}\left[ \left( \sum _{p=3}^{r}\sigma _{p}X_{mn}^{\left( p\right) }\right) \exp \left( -\frac{ 2\pi ink}{r}\right) \right] \right| ^{2}. \end{aligned}$$

Clearly, $F\left( k\right)$ measures the averaged Fourier weight of the new eigenvalue spectra $\sum _{p=3}^{r}\sigma _{p}X_{mn}^{\left( p\right) }$, which are the spectra after dropping the first two components. And the second one, which bears analytical treatments^49–51^, is

6 $$\begin{aligned} S\left( k\right) =\frac{1}{N}\sum _{i=1}^{N}\left| \delta _{k}^{\left( i\right) }\right| ^{2} \end{aligned}$$

where

7 $$\begin{aligned} \delta _{k}^{\left( i\right) }= & {} \frac{1}{N}\sum _{n}\delta _{n}^{\left( i\right) }\exp \left( -\frac{2\pi ikn}{N}\right) \text {,} \nonumber \\ \delta _{n}= & {} \sum _{i=1}^{n}\left( s_{i}-\langle s\rangle \right) =s_{n}-n\langle s\rangle \text {.} \end{aligned}$$

**Figure 3:**
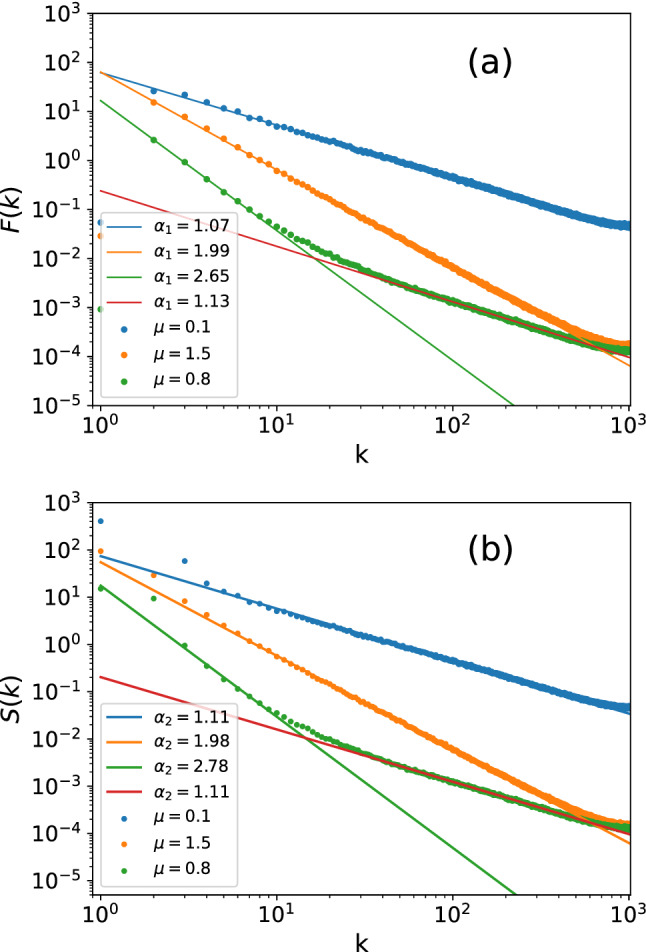
Two kinds of power-spectrum functions—*F*(*k*) in Eqs. (4), (5) and *S*(*k*) in Eqs. (6) and (7), they show very similar scaling behaviors to $\lambda _k$ shown in Fig.1b, suggesting that they contain essentially identical information.

The $S\left( k\right)$ is the averaged Fourier weight of the new cumulated level spacing $\left\{ \delta _{n}\right\}$. The numerical results of $F\left( k\right)$ and $S\left( k\right)$ are shown in Fig. 3, we see that their scaling behaviors are totally alike to $\lambda _{k}\left( k\ge 3\right)$, that is, $F\left( k\right) \sim k^{-\alpha _{1}}$ and $S\left( k\right) \sim k^{-\alpha _{2}}$ with $\alpha \simeq \alpha _{1}\simeq \alpha _{2}$ in all regimes, which indicates $F\left( k\right)$ and $S\left( k\right)$ essentially contain identical information with $\lambda _k$. This result is consistent with our earlier analysis about $W_k$, and the detailed analysis goes as follows.

We have argued that $W_{k}$ is close to the $k-\hbox {th}$ Fourier mode of the eigenvalue spectrum for $k\ge 3$, hence the power-law behavior $\lambda _{k}\sim k^{-\alpha }$ essentially stands for a decreasing trend of the eigenvalues’ Fourier weights. On the other hand, the definition of $F\left( k\right)$ in Eqs. (4), (5) drop the first two dominant terms $X^{\left( 1/2\right) }$, which stand for the mean energy $\langle E\rangle$ and level spacing $\langle s\rangle$. Since $\langle E\rangle$ and $\langle s\rangle$ are both non-fluctuating, the fluctuating behaviors of original eigenvalue spectra and the new one ($\sum _{p=3}^{r}\sigma _{p}X_{mn}^{\left( p\right) }$) should be the same, which is reflected by the similarity between $\lambda _k$ and *F*(*k*) in Fig. 3. The same arguments apply to *S*(*k*) as well. From *S*(*k*)’s construction in Eq.(6)-(7), it’s easy to see the information of $\langle E\rangle$ and $\langle s\rangle$ are both lost, consequently, its scaling behavior should also be similar to $\lambda _k$, in the same sense as $F\left( k\right)$.

Having uncovered the physics of $W_{k}$, we’re ready to understand the meaning of the two-branch scree plots in Fig. 1b: it simply indicates the eigenvalue correlations on short and long ranges behave *qualitatively* different in this regime, which is a fingerprint of the NEE regime. Moreover, the appearance of the super-Poissonian behavior $\lambda _{k}\sim k^{-\alpha }$ is consistent with miniband picture of NEE regime, in the same sense as it does in the RP model^29,30^. Combining these arguments, the region of Fig. 1b is confirmed to represent the NEE regime. More importantly, we see that the starting point of the super-Poissonian behavior is very close to the predicted value $\mu _{c}\simeq 0.5$ in Ref.^40^, indicating the scree plot is accurate in identifying the ergodic-NEE transition point, although the estimation for the NEE-MBL transition point is less accurate. This which makes SVD a more powerful tool in studying NEE physics, for which we will provide further evidence in the next section.

Before proceeding, there’re two technical issues need clarifying. Firstly, the reason for selecting only the middle part of eigen-levels to do SVD is to avoid confusions raised by the (possible existence of) mobility edge, that is, eigenstates in different part of the spectrum may belong to different phases which results in mixed scaling behaviors of $\lambda _{k}$. Second issue is about the choice of *N*/*P*. We have verified that when *N*/*P* is too large ($N/P>1$), the scree plots will have rapidly decreasing tails that are insignificant, such a situation have also been noted in previous studies^30,31^; while when *N*/*P* is too small, the number of singular values is too small to reveal clear power-law scaling. For these practical reasons, we keep $N/P=1/2$ throughout this study.

## Scree plots of other RM models

In this section we employ SVD to three different random matrix (RP) models: (i) the RP model, where a NEE is known; (ii) a sparse RM that describes percolation between GOE and Poisson^52^, we will identify a new NEE regime; (iii) Gaussian $\beta$ ensemble, which contains no NEE with positive $\beta$.

The RP model is the first RM model that analytically proved to hold a NEE regime^39,42^, which is later justified through SVD^30^. Here we present a more detailed study to show the ergodic-NEE transition point can also be accurately identified through SVD, just like in the PRBM case in previous section. Specifically, the RP model is a random matrix whose non-diagonal terms following $N\left( 0,N^{-\gamma }/6\right)$ and diagonal terms $N\left( 0,1\right)$, this fixes $\langle H_{ij}^{2}\rangle /\langle H_{ii}^{2}\rangle =N^{-\gamma }/6$, and the NEE regime is predicted to be $\gamma \in [1,2]$^39,42^. To do SVD, we likewise generate $N=1000$ samples of eigenvalue spectra with matrix dimension $D_{H}=10{,}000$, and take out the middle 2000 eigenvalues to construct the sample matrix *X*.

**Figure 4:**
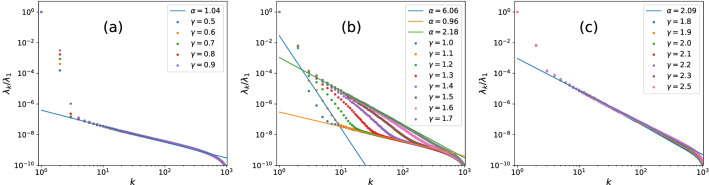
Scree plots of the RP models at various parameters $\gamma$ , the middle figure (**b**) stands for the NEE regime, identified through the two-branch structure with super-Poissonian behavior $\lambda _k\sim k^{-\alpha },\alpha >2$. The starting point of NEE regime is $\gamma =0.9\sim 1$, consistent with predicted value $\gamma _c\simeq 1$^39,42^.

The resulting scree plots for the RP model at various $\gamma$s are displayed in Fig. 4, we see it divides into three categories just like the PRBM case in Fig. 1. More precisely, the cases with $\gamma <1$ display overall ergodic behaviors with $\lambda _k\sim k^{-1}$ for $k\ge 3$. While clear suer-Poissonian behavior starts to appear when $\gamma$ approaches to $\gamma =0.9\sim 1$. While when $\gamma >1.7$ it enters into the fully chaotic regime with $\lambda _k\sim k^{-2}$. According to the semi-analytical predictions^39,42^, the NEE regime for RP model is $1<\gamma <2$, therefore the scree plot can accurately identify the ergodic-NEE transition point, while its estimation for the NEE-MBL transition is less accurate, this is consistent with the results for PRBM in previous section.

**Figure 5:**
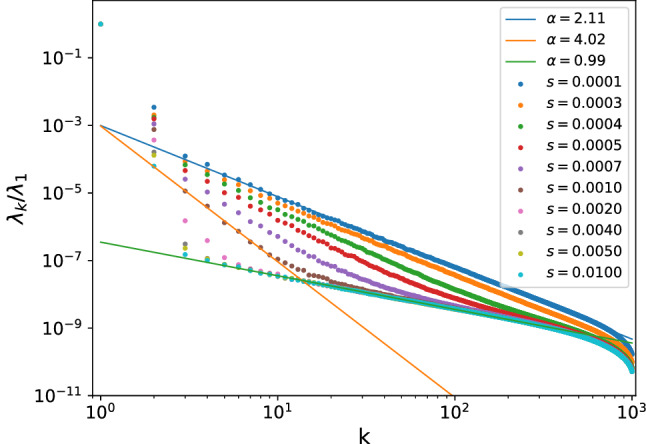
Scree plots of the sparse RM. Clear super-Poissonian behaviors appear, indicating there is NEE regime in this model as well, and the ergodic-NEE transition point is identified to be $s_c\simeq 0.005$.

Having shown the SVD can accurately identify the ergodic-NEE transition point, it is natural and tempting to employ it to discover NEE regime in unknown RM models. Here we report such an example, that is, the sparse RM model^52^ that also describes the interpolation between Wigner–Dyson and Poisson. This model is defined through the sparsity parameter *s*, which is the fraction of the non-zero off-diagonal elements at random positions that follow the standard Gaussian distribution $N\left( 0,1\right)$, while the diagonal elements follow $N\left( 0,\sqrt{2}\right)$. In the limit $s\rightarrow 1$ it recovers to the standard Wigner–Dyson ensemble, and in $s\rightarrow \infty$ it reduces to Poisson ensemble. To do SVD, we keep $N=1000$ and $D_H=8000$ for numerical simulation, and take out middle $P=2000$ eigen-levels as before, the scree plots at various sparsity are given collectively in Fig. 5.

As can be seen, when the two-branch structure with super-Poissonian behavior exists in the range $s=\left[ 10^{-4},10^{-2}\right]$, or equivalently $\log _{10}s=\left[ -4,-2\right]$, indicating that NEE regime also exists in this RM model. More precisely, the super-Poissonian behavior starts to appear at roughly $s_c=0.005$, which is hence identified to be the ergodic-NEE transition point. When $s<0.0001$ it enters into the fully chaotic phase, while when $s>0.01$ fully integrable behaviors appear.

Having observed NEE regimes in aforementioned three RM models, it’s natural to guess that the super-Poissonian behavior generically exists in any model that interpolates between Wigner–Dyson and Poisson. However, we shall see that it is not the case. To show this, we study the Gaussian $\beta$ ensemble, whose joint-probability distribution follows the same form as Wigner–Dyson classes while the level repulsion parameter $\beta$ takes continuous value in $\left( 0,\infty \right)$, i.e.

$$\begin{aligned} P\left( \beta ,\left\{ E_{i}\right\} \right) =C\prod _{i<j}\left| E_{i}-E_{j}\right| ^{\beta }e^{-A\sum _{i=1}^{N}E_{i}^{2}}\text {, }\beta \in \left( 0,\infty \right) \text {.} \end{aligned}$$

**Figure 6:**
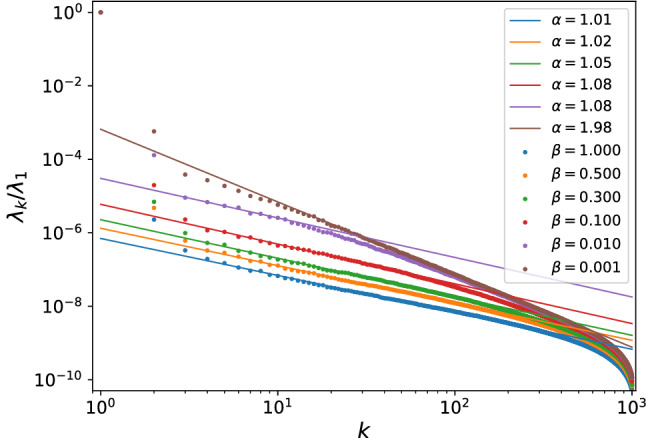
Scree plots of Gaussian $\beta$ ensemble with $\beta \in (0,1]$, chaotic behavior $\lambda _k\sim k^{-1}$ maintains in the whole range $\beta \in (0.01,1]$. The Poissonian behavior $\lambda _k\sim k^{-2}$ only occurs when $\beta$ is extremely small, where the eigenvalues are practically uncorrelated with finite matrix dimension and numerical accuracy. In none case does the super-Poissonian behavior appear, suggesting no NEE regime exists.

As we can see, the eigen-levels maintain full-range logarithmic correlation for however small $\beta >0$, so the system maintains ergodic for any positive values of $\beta$. In other words, this model displays an *abrupt* ergodic-integrable transition at $\beta _c=0$, no NEE is expected with tuning positive $\beta$, and its scree plots should contain no super-Poissonian behaviors. To verify this, we likewise collect the sample matrix of $\beta$ ensemble, whose construction is totally alike to the aforementioned sparse RM model. Here the eigenvalue spectra can be efficiently obtained by diagonalizing the following tri-diagonal matrix^53^

8 $$\begin{aligned} M_{\beta }=\frac{1}{\sqrt{2}}\left( \begin{array}{ccccc} x_{1} &{} y_{1} &{} &{} &{} \\ y_{1} &{} x_{2} &{} y_{2} &{} &{} \\ &{} \begin{array}{ccc} \text {.} &{} &{} \\ &{} \text {.} &{} \\ &{} &{} \text {.} \end{array} &{} \begin{array}{ccc} \text {.} &{} &{} \\ &{} \text {.} &{} \\ &{} &{} \text {.} \end{array} &{} \begin{array}{ccc} \text {.} &{} &{} \\ &{} \text {.} &{} \\ &{} &{} \text {.} \end{array} &{} \\ &{} &{} y_{N-2} &{} x_{N-1} &{} y_{N-1} \\ &{} &{} &{} y_{N-1} &{} x_{N} \end{array} \right) \end{aligned}$$

where the diagonals $x_{i}\,$ follow the normal distribution $N \left( 0,2\right)$ and $y_{k}$ ($k=1,2,\ldots ,N-1$) follows the $\chi$ distribution with parameter $\left( N-k\right) \beta$. The resulting scree plots for $\beta$ ensemble are given in Fig. 6.

As expected, the scree plots show no super-Poissonian behaviors in the whole range $\beta \in (0,1)$. The majority of $\lambda _{k}$ follows a clear chaotic behavior $\lambda _{k}\sim k^{-1}$ for $\beta \in$ (0.1,1]. When $\beta$ decrease to 0.01, the scree plots begin to hold chaotic behavior ($\alpha =1$) and integrable behavior ($\alpha =2$) at the same time, this is because the matrix dimension we consider is finite ($D_H=8000$), hence when $\beta$ is too small, the eigenvalues are close to be independent within numerical accuracy. When $\beta$ is so small as 0.001, it practically becomes a Poisson ensemble. Nevertheless, no sign of NEE regime ever appears, which is consistent with our earlier analysis.

Before concluding, we want to mention that the Gaussian $\beta$ ensemble is not the only model that describes the interpolation between Wigner–Dyson and Poisson while contains no super-Poissonian behavior. Another example is the one-parameter RM model proposed by Seligman et al. in Ref.^54^, which is a Gaussian orthogonal ensemble (GOE) with the matrix elements $H_{ij}$ multiplied by a factor $e^{-|i-j|/\epsilon ^2}$. Clearly, this RM model recovers to GOE/Poisson in the limit $\epsilon \rightarrow \infty /0$, its scree plots are reported in Ref.^23^ that contains no super-Poissonian behaviors with changing $\epsilon$, which indicates that NEE regime does not exist in this RM model either.

## Conclusion and discussion

We have employed the method of SVD to study the eigenvalue spectra of several typical random matrix models, and reached the following important conclusions: (i) the scree plots $\lambda _{k}$ with super-Poissonian behavior $\lambda _{k}\sim k^{-\alpha }$ with $\alpha >2$ is a fingerprint of NEE regime; (ii) the point at which the super-Poissonian behavior begins to appear gives an accurate identification for the ergodic-NEE transition point; (iii) the existence of NEE regime in a new random matrix model—the sparse random matrix—is suggested, where the ergodic-NEE transition point is identified to be $s_{c}\simeq 0.005$.

We also show the super-Poissonian behavior is absent in the Gaussian $\beta$ ensemble, and point out the same situation also appears in the one-parameter RM model proposed by Seligman^23,54^, which suggest the absence of NEE in these models. Therefore, a general criteria for the existence of absence of NEE regime is a crucial question, which will be left for future studies.

Compared to conventional approaches to study NEE regime, the method of SVD has two outstanding advantages: (i) it requires only the eigenvalue spectra, which is technically much easier to obtain than the eigenstate wavefunctions; (ii) it does not require unfolding procedure as it does when studying number variance or spectral form factor, which makes it free of potential unambiguity raised by concrete unfolding strategy.

Another advantage of SVD is that it’s highly generalizable, it is in principle applicable to any quantum systems with eigenvalue spectra. For example, the non-Hermitian systems with complex eigenvalues, it’s interesting to explore whether the power-law scree plots exist in non-Hermitian systems or not, which is also a promising future direction.

Finally, as stated in the “Introduction” section, the spirit of SVD is to view the eigenvalue spectra as a multi-variant time series, it is thus natural to ask what the scree plot would be in the sample matrix of real-life time series, for example, the prices of stocks, where the RMT has already been proved to be a useful tool^55–59^. We have actually tested SVD in such a system, which is presented in the supplementary material S1.

## Supplementary Information

Supplementary Information.

## Data Availability

The data sets used and/or analysed during the current study are available from the corresponding author on reasonable request.
