# Supplementary material for: Non-ergodic extended regime in random matrix ensembles: insights from eigenvalue spectra
Source: Sci Rep. 2023 Jan 12;13:634. doi: 10.1038/s41598-023-27751-9 (PMC9837147; doi:10.1038/s41598-023-27751-9)
Supplement: Supplementary file 1 — Supplementary Information. [file 41598_2023_27751_MOESM1_ESM.pdf]

# Supplementary material to
# Non-Ergodic Extended Regime in Random Matrix Ensembles: Insights from Eigenvalue Spectra

Wang-Fang Xu[1,2] and W. J. Rao*[1]

[1]*School of Science, Hangzhou Dianzi University, Hangzhou 310027, China.*
[2]*China Academy for Rural Development & School of Public Affairs, Zhejiang University, Hangzhou 310027, China.*
(Dated: November 3, 2022)

## I. SCREE PLOT OF EMPIRICAL DATA FROM STOCK MARKET

The usage of random matrix theory in analyzing stock prices dates back to more than twenty years ago, where one typically collect the pricing series of a large number of companies to construct the correlation matrix, and the majority of the eigenvalues follows the prediction of random matrix theory, with rare exceptions reflecting truly non-trivial information (see, for instance, Ref.[53-57]). Here we employ SVD to analyze the data from stock market. Specifically, we collect the daily prices of $444$ companies in S&P500 index during the time range 2010-11 to 2020-11, where each company contains a price series of $2544$ trading days, the data are downloaded from Yahoo Finance. Following the standard strategy, we use the normalized price change as the input data, then all the data are arranged into a sample matrix $X$ of dimension $N \times P = 444 \times 2543$. Then we perform SVD to get $X = U^T \Lambda V$, the scree plots $\lambda_k$ now appears in Fig. SM1(a).

As we can see, now the first two weights $\lambda_{1/2}$ are not so dominant as in the RM models in the main text. Most interestingly, we see the $\lambda_k$ also presents a two-branch structure, but now the lower-part $(2 < k < 20)$ displays an chaotic behavior $\lambda_k \sim k^{-1}$, while the higher-part follows $\lambda_k \sim k^{-\alpha}$ with $\alpha \simeq 0.54 < 1$, in sharp contrast to the RM models studied in the main text. According to the discussions in the main text, this may hint the correlations between companies follows a typical GOE in long time scale, while in shorter time scales it remains unclear.

On the other hand, the common wisdom of previous works is that the eigenvector of the correlation matrix with largest eigenvalue displays a close-to-uniform distribution, standing for a collective motion of all companies. This can also be verified through SVD, where the interactions between companies are now represented by the columns of matrix $U^T$ , or equivalently $U_k$ – the $k$-th row of $U$ – with weight $\sigma_k$. We then draw the first three $U_k$ in Fig. SM1(b). Clearly, the first component $U_1$ is close to a constant value around $0.05$, which represents the uniformly collective motion of all companies, in accordance with previous studies. Higher components $U_k$ $(k > 1)$ display fluctuations around $0$, a detailed analysis may reflect the sector structures of the stock market, which, together with the explanations for the observed scree plots, will be exploited in a future work with larger data sets.

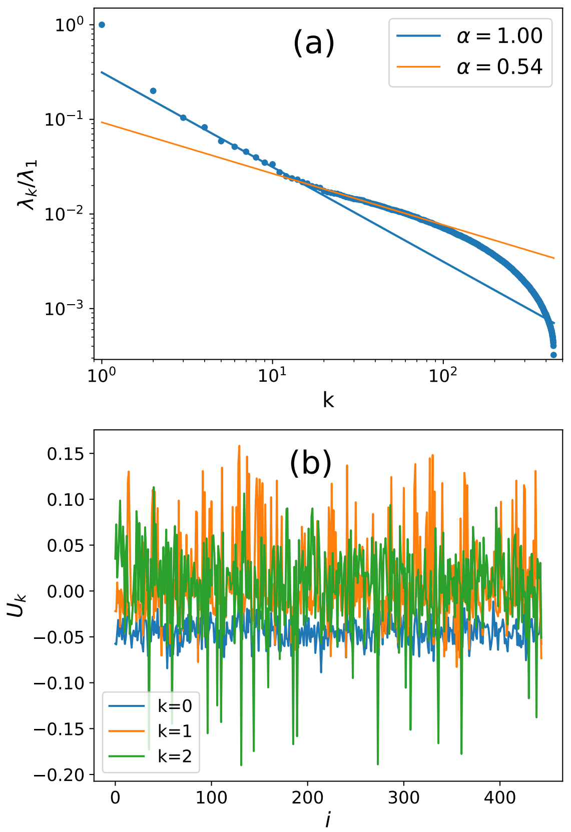


FIG. SM1. (a) Scree plot of the empirical sample matrix comprised of stock price changes. Interestingly now the lower part follows a chaotic behavior, while higher part displays novel behavior $\lambda_k \sim k^{-\alpha}$ with $\alpha < 1$. (b) Distributions of first three components $U_k$, the dominant $U_1$ is close to a uniform distribution, which stands for a collective motion of all companies, in consistent with earlier studies[?,?].
